# Optimization of Genome Knock-In Method: Search for the Most Efficient Genome Regions for Transgene Expression in Plants

**DOI:** 10.3390/ijms23084416

**Published:** 2022-04-16

**Authors:** Sergey M. Rozov, Natalya V. Permyakova, Yuriy V. Sidorchuk, Elena V. Deineko

**Affiliations:** Laboratory of Plant Bioengineering, Institute of Cytology and Genetics, Siberian Branch of Russian Academy of Sciences, 630090 Novosibirsk, Russia; puh@bionet.nsc.ru (N.V.P.); sidorch@bionet.nsc.ru (Y.V.S.); deineko@bionet.nsc.ru (E.V.D.)

**Keywords:** recombinant proteins, actively transcribed regions, plant expression systems, housekeeping genes

## Abstract

Plant expression systems are currently regarded as promising alternative platforms for the production of recombinant proteins, including the proteins for biopharmaceutical purposes. However, the accumulation level of a target protein in plant expression systems is still rather low compared with the other existing systems, namely, mammalian, yeast, and *E. coli* cells. To solve this problem, numerous methods and approaches have been designed and developed. At the same time, the random nature of the distribution of transgenes over the genome can lead to gene silencing, variability in the accumulation of recombinant protein, and also to various insertional mutations. The current research study considered inserting target genes into pre-selected regions of the plant genome (genomic “safe harbors”) using the CRISPR/Cas system. Regions of genes expressed constitutively and at a high transcriptional level in plant cells (housekeeping genes) that are of interest as attractive targets for the delivery of target genes were characterized. The results of the first attempts to deliver target genes to the regions of housekeeping genes are discussed. The approach of “euchromatization” of the transgene integration region using the modified dCas9 associated with transcription factors is considered. A number of the specific features in the spatial chromatin organization allowing individual genes to efficiently transcribe are discussed.

## 1. Introduction

Currently, recombinant proteins are widely used in medicine and veterinary as well as in other areas of human activities. First and foremost, this includes vaccines, monoclonal antibodies, drugs, diagnostic tools, and so on [1]. Recombinant proteins are synthesized in prokaryotic and eukaryotic expression systems, such as Escherichia coli, yeast, insect cells, and mammalian cell cultures. Over half of all pharmaceutical proteins are produced in mammalian cells [2,3], since the prokaryotic systems and yeasts are incapable of certain posttranslational modifications (PTMs) characteristic of eukaryotes [4,5]. Incorrect PTMs or their absence can considerably change the properties of a synthesized protein, including its biological activity and pharmacokinetics. Thus, the prokaryotic expression systems are currently used mainly for synthesizing relatively simple therapeutic proteins, while the proteins that are more complex are frequently produced in the expression systems involving mammalian cells [2,3]. However, even the latter have their flaws, in particular, rather expensive cultivation, difficulties with upscaling of the process, and potential viral contamination. Although there are some examples of the successful use of plant suspension cell cultures in commercial production of valuable proteins, the number of yet unsolved problems in this area is still rather large. The most important problem is an insufficient yield of the recombinant protein in plant cells, which rarely exceeds 100 µg/kg biomass [4,5].

The currently available technologies for the delivery of foreign genes to the plant genome are mainly based on the random insertion into transcriptionally active or transcriptionally inactive regions. It is thus evident that implementation of the potentially high expression level of the target genes maximally optimized by researchers directly depends on the random distribution of foreign DNA insertions in the genome. Correspondingly, the expected optimally high expression level of a transgene may well be unattained if the transgene finds itself inserted into transcriptionally inactive genomic regions. This particular fact underlies the observed variation in the expression level of transgenes among the individually constructed transgenic plants [5]. The existing technologies for the production of recombinant proteins in plant expression systems comprise the routine stage of selection of the most favorable transformation events associated with a high yield of the recombinant protein [6].

The development of molecular biological and genetic engineering methods and their enhancement as well as the rapid development of the genome editing techniques utilizing CRISPR/Cas allows researchers to set forth the targeted gene delivery to almost any selected constitutively transcribed genomic regions. This will further make it possible to dispense with the laborious screening of a large number of transgenic lines to purposefully construct highly efficient producers of recombinant proteins carrying the target genes delivered to transcriptionally active regions. The site-specific endonucleases (Cas9 being the best known) can make double-strand DNA breaks (DSBs) in a specified genomic region, which can be further repaired according to one of the two main mechanisms existing in eukaryotes: nonhomologous end joining (NHEJ) or homologous DNA repair (HDR). The repair in all eukaryotes except for yeasts prevalently follows one of the variants of NHEJ pattern [7,8]. If a template carrying a transgene is present nearby during the repair, there is a certain probability that this transgene will be knocked-in in a DSB, providing the insertion of an additional gene into the specified genomic region [9,10,11,12].

It becomes obvious that the modern method of delivering target genes to specific regions of the genome using the CRISPR/Cas system should be accompanied by preliminary work to identify the regions that are most optimal for targeting editing tools. This approach has opened up the possibility of avoiding some negative phenomena associated with the conventional transgenesis, such as variability in target gene expression, T-DNA-induced mutations, and gene silencing. This has stimulated researchers to search for suitable regions in the plant genome for site-specific delivery of target genes. In particular, researchers seek the regions of the genome, the so-called genomic "safe harbors", where no changes in any agronomic character occur upon delivery of one or more linked target genes. The identification of genomic “safe harbors” is becoming a reality due to the development of high-throughput phenotyping methods [13,14].

The attractiveness of this approach, i.e., the preliminary selection of the target region for the delivery of the target gene, is that the researcher assesses the “risks” of delivering the target gene into the plant genome in terms of obtaining the final product. That is, the improvement of one trait by integration into the genome of the target gene should not change or impair the expression of other important characteristics of the improved plant variety. The successful application of this approach was implemented in the modification of some agricultural crops, for example, in the creation of golden rice [15].

In the case of using plants as bioreactors for the production of recombinant proteins, the problem of finding the most optimal regions for the delivery of target genes also remains extremely relevant. The main criterion for choosing such regions should be not only a high level of expression of the target gene but also a high yield of the target recombinant protein in the expression system used, for example, in plant cell culture. The authors of this review consider areas of housekeeping genes for plant cell cultures as very promising for these purposes [16,17]. Compared with random insertion, site-specific insertion into the region of housekeeping genes makes it possible to obtain high and stable expression of the target gene with a high probability. These regions are attractive because the housekeeping genes are actively expressed during the entire interphase of the cell cycle, providing the synthesis of vitally important cell proteins. The copy number of housekeeping genes in the genome is very high, and they mainly reside in euchromatic genomic regions [18]. The organization and regulation of the transcription and translation machinery, having formed during a long evolution, ensure the stable operation of these genes and a constitutive pattern of protein synthesis. In addition, loci directly adjacent to the housekeeping genes or their intergenic spacers can be chosen in such way to serve simultaneously as a "safe harbor" of the genome for the insertion of target genes. Thus, the detection of the transcriptionally active genomic regions, in particular, the regions harboring housekeeping genes, and targeted integration of genes into these regions may open new vistas for an increase in the synthetic capabilities of plant cells in producing recombinant proteins.

In this regard, the main purpose of this review is the improvement of the knock-in method of genome editing by the selection of optimal regions for targeted integration of genes into the plant genome. Examples of successful solutions to some problems of target gene delivery to genomic “safe harbors” are given. Various approaches are considered aimed at identifying regions of the genome characterized by high transcriptional activity that could serve as targets for the delivery of target genes using CRISPR/Cas9 and could be used in biotechnology to create highly productive cell lines producing recombinant proteins. An analysis of modern approaches aimed at increasing transcriptional activity or “euchromatization” of the region of transgene integration was carried out. In addition, the specific features in a spatial organization of chromatin that allow individual genes to transcribe most intensively are also considered.

## 2. Genomic Safe Harbors

The term “genome safe harbors” (GSH) was first used in the development of functional genetic studies in human gene therapy. This term denotes regions in the genome where the integration of a new genetic material ensures its functional predictability and does not cause negative changes in the host genome, such as, for example, malignant clonal expansion of human cells [19]. GSH can be considered as the “ideal” genome regions for the integration of transgenes into them, and their search is an indispensable part of the work associated with the transfer and targeting of transgenes to these regions. It is the possibility of delivering target genes to certain regions of the genome using the CRISPR/Cas system that makes it necessary for researchers to search for GSH as well as to develop criteria for their safety. Such criteria need to be developed, not only for the field of human gene therapy, but also for other areas of research, such as cultured cell biotechnologies or agrobiotechnologies using targeted DNA insertion into the plant genome.

Let us consider the main GSH search criteria used by various groups of researchers in solving a specific scientific problem related to the delivery of target genes to specific regions of the genome using the CRISPR/Cas system. On the model of thalassemia disease, several criteria were determined for the region of integration of the gene encoding β-globin into the human genome, compliance with which ensured the level of expression of the target protein (β-globin) required for the manifestation of a therapeutic effect and did not disrupt the regulation of endogenous gene expression [20]. For human cells, the three most reliable GSHs were found: AAVS1, the natural integration site of the AAV virus on chromosome 19; CCR5, chemokine receptor 5, known as the HIV-1 co-receptor; and the human ortholog of the mouse Rosa26 locus [21]. Currently, these GSHs are successfully used in gene delivery studies targeting these regions [22]. A software package for computer prediction and experimental verification of GSH in the human cell genome has been developed [23]. Using the example of two genomic sites, Rogi1 and Rogi2, the authors of this software package demonstrated the success of using these sites as GSH for the expression of target genes in various contexts of the genome. Further prospects in the direction of optimizing the GSH search criteria and testing their functional characteristics in human gene therapy are considered in reviews [21,24].

To improve the nutritional characteristics of cultivated plant varieties, methods of genetic engineering and genome editing are now widely used. The main criteria that must be taken into account when carrying out this kind of work are the preservation of the characteristics of the variety created by the previous efforts of breeders. These characteristics include productivity, resistance to adverse environmental factors, resistance to insect pests, viral and bacterial diseases, lack of moisture, etc. It is these characteristics that were taken by researchers as the main ones when inserting target genes into specific regions of the genome using the CRISPR/Cas system in studies to improve the nutritional value of rice grain by increasing its carotenoid content [15]. Among the induced mutations in the rice variety Kitaake, five GSHs were isolated and characterized using high throughput phenotyping techniques. Studies have shown that mutations in these five regions did not adversely affect the studied characteristics of rice, and two of them have been successfully used to deliver carotenoid biosynthesis genes [15].

In the field of biotechnology, the production of recombinant proteins, including biopharmaceutical ones, is based on methods for transferring genes from various heterologous systems into the genomes of bioproducing cells [25]. The features of the cultivation of cells induced from tissues of multicellular organisms in bioreactors are that most of the genome encoding the functioning of a multicellular organism is not in demand under in vitro conditions [26,27]. Therefore, the search for sites to deliver target genes via the CRISPR/Cas system should be based on criteria other than those listed above. For example, one of the most important unsolved problems for using plant cell cultures as bioreactors is the still low yield of recombinant proteins in these expression systems. Thus, the search for regions to direct the insertion of target genes into the regions of the genome with an optimally high level of transcriptional activity can serve as the main criterion for such a search. In this case, the regions of housekeeping genes characterized by high transcriptional activity can serve as the most suitable candidates to insert target genes.

## 3. Housekeeping Genes

A high and stable expression of a target region in the plant genome requires the gene to be situated in a region with high transcriptional activity. The genomic regions containing the so-called housekeeping genes, constitutively and actively expressed through the interphase of the cell cycle, are the most appropriate for this purpose. In addition, it is preferable that such a gene is contained in the genome in several copies, which makes it possible to insert several copies of a transgene in a targeted manner. The set of genes meeting these conditions is rather large; so, we will consider only the most promising of them. First and foremost, these are the genes coding for different RNAs involved in protein synthesis (35S pre-rRNA, 5S rRNA, and tRNA), histone genes, and the genes coding for actin, tubulin, and ubiquitin. Table 1 shows the data on their copy numbers in the *A. thaliana* genome, their organization, chromosome localization, specific transcriptional features, and the phase of activity in cell cycle [5].

### 3.1. 35S (45S) Pre-rRNA Genes

In the nuclear genome of higher plants, the genes coding for 18S, 5.8S, and 25S rRNAs are organized in a single transcriptional unit with a length of ~5 kbp and are separated by internal transcribed spacers (ITSs) [28]; by the analogy with the animal rRNA genes, this unit is frequently referred to as the 45S rRNA genes. Actually, this single transcript is shorter because of shorter spacers and has a sedimentation coefficient of 35S [29]. The copy number of these operons in the plant genome ranges from a thousand to tens of thousands; they form long tandem repeats located in one or several loci. The transcriptional units in such repeats are separated by more variable untranscribed intergenic spacers (IGSs) with a length of 5–15 kbp [30]. The 35S pre-rRNA genes are transcribed by RNA polymerase I and form the so-called nucleolar organizer region (NOR), which forms the nucleolus. Undoubtedly, this is the most transcriptionally active region in the plant cell, and from this point of view it looks very attractive for the insertion of target genes.

However, not a single event of the gene delivery to a selected target region of the nucleolar organizer (in total, several hundred attempts) has been recorded in the experiments on the delivery of different genetic constructs using both biolistics and agrobacterial transformation [17].

**Figure 1:**
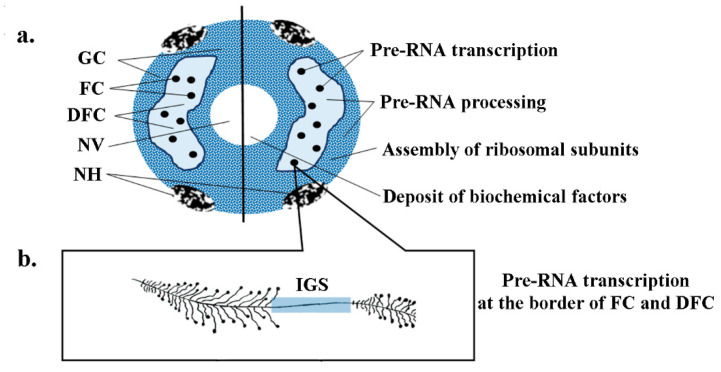
General scheme of the nucleolus and the local regions of plant rRNA transcription: (**a**) nucleolus structure (GC, granular component; FC, fibrillar center DFC, dense fibrillar component; NV, nucleolar pseudo-vacuole; and NH, nucleolus-associated heterochromatin); and (**b**) ribosomal DNA with multiple transcription sites and intergenic spacer (gray).

As has recently emerged, the nucleolus is an amorphous structure far from being uniform but rather split into distinct compartments, nucleolonemes, with the transcription centers containing decondensed heterochromatin [31,32,33]. Figure 1 schematizes the structure of the nucleolus in the plant cell nucleus and the processes taking place there (for details of the nucleolus structure, see reviews [31,32,33].

The main structural components of the nucleolus detectable using an ultrastructural examination (Figure 1a) are fibrillar centers (FCs), dense fibrillar component (DFC), granular component (GC), nucleolar vacuole (NV), and the heterochromatin associated with the nucleolus (NH). All these components have their own functions. For example, rDNA is transcribed at the boundary between fibrillar centers and the dense fibrillar component, which occupies the larger part of the plant nucleolus volume. The pre-rRNA can be concurrently transcribed from several rDNA regions (Figure 1b) and is processed in the dense fibrillar component; then, the last stages in the assembly of small and large ribosomal subunits of the mature rRNAs and ribosomal proteins take place in the granular component (Figure 1a).

The most recent data on the organization of the nucleolus and the processes occurring in it suggest that this region is extremely problematic for the insertion of exogenous DNA independently of the methods used for its delivery to the cell. First and foremost, this is associated with the specific features in the organization of the nucleolus as well as an extremely high level of transcriptional and posttranscriptional activities. This is explainable with the fact that the constantly formed products, such as pre-rRNA, ribonucleoprotein complexes, and ribosomal subunits, along with ribosomal proteins, create an insurmountable mechanical barrier for the delivery of exogenous DNA. In addition, the presence of RNA polymerase II, necessary for the transcription of the genetic constructs carrying the genes to be translated, in the nucleolus is still a matter of dispute [31,32,34].

In addition to the specific structure–function features of the nucleolus, the repair systems that provide the stability and preservation of rRNA genes are at least an equal hindrance for the knock-in in this region. A high level of transcription along with a high copy number of rRNA gene repeats makes it prone to the emergence of double-strand breaks [35]. DSBs are serious damages interfering with DNA replication and transcription with the potential to cause significant chromosome rearrangements threatening cell viability. The need to defend the rRNA genes from the consequences of DSBs has led to the emergence of the so-called nucleolar caps (nucleolar heterochromatin), the unique structures in the periphery of the nucleolus where the damaged (carrying DSBs) regions of rRNA genes are moved. Approximately half of the rRNA gene copies are silent and reside in the perinucleolar heterochromatin [35]. Consequently, even if a sufficient number of DSBs is present in the nucleolar organizer region, any possibility to use this region as the target for the genes is excluded, not only because their delivery there is difficult, but also because the delivered construct with a high probability finds itself in a silent perinucleolar heterochromatin.

Thus, although the region of ribosomal genes is most attractive for the targeted insertion of genetic constructs requiring a high transcription level, this region does not seem available for Cas9, donor vectors and RNA polymerase II because of its intricate compartmentalization, clarified most recently, and is unsuitable for genome editing.

### 3.2. 5S rRNA Genes and tRNA Genes

The genes of 5S rRNA in the majority of eukaryotes are located separately from the 35S rRNA gene cluster. Nonetheless, the 5S rRNA genes of yeasts, some nematodes and arthropods, and some plants (part of the Asteraceae family and some gymnosperms) reside in 35S rRNA tandem repeats within IGSs and are transcribed by RNA polymerase III in either a forward or a reverse direction [29,36]. The diploid genome of *A. thaliana* contains ~2000 copies of 5S rRNA genes [37] organized in long tandem arrays in the pericentromeric regions of chromosomes 3, 4, and 5. Chromosomes 3 and 4 carry one 5S rRNA gene locus each, while chromosome 5 has a large 5S rRNA locus in its one arm and a small locus in the other. The locus in chromosome 3 is rather small and is detectable not in all ecotypes; its position in the chromosomes is rather variable [38]. The repeated unit of 5S rRNA genes contains a transcribed region of ~120 bp and an IGS with a length of ~480 bp. Only the genes in chromosome 4 and in the large locus of chromosome 5 are expressed. In total, they comprise ~300 genes (haploid genome) coding for two classes of 5S rRNA, the major class (82%) and the minor class (18%), which differ in 1–2 nucleotide substitutions in the transcribed region. At the initial stages of shoot development, the share of the minor class may reach 25% [39,40].

The nuclear genes of the transport RNAs of plants, as well as of other eukaryotes, are multigenic families where individual copies are either disorderly scattered through the genome or clustered in one or several sites. The first variant is more typical of the plant tRNAs. The majority of tRNA genes are actively and constitutively transcribed during the entire interphase of the cell cycle. Usually, the plant genome contains 10–20 copies of the genes of each tRNA. For example, the *A. thaliana* genome contains at least 20 copies of the gene coding for tRNA^Tyr^ and the *Nicotiana rustica* genome at least 14 copies. All copies of the tRNA^Tyr^ gene detected in *A. thaliana* are organized as long tandem repeats with a repeated unit 1.5 kbp long; note that this unit also contains the gene of tRNA^Ser^ [41,42]. Similar to 5S rRNA, the tRNA genes are transcribed by RNA polymerase III [43].

Neither 5S rRNA genes (if they reside beyond the 35S rRNA cluster) nor the tRNA genes of plants form any dense physical structures like the nucleolus and are thus potentially available for the CRISPR/Cas9 tools and RNA polymerase II. The intergenic spacers in the case of tandemly repeated genes of the knowingly euchromatic regions flanking the gene are the promising sites for the insertion of target recombinant genes into the regions of plant 5S rRNA and tRNA genes.

### 3.3. Histone Genes

The arrangement of histone genes considerably differs in different groups of living organisms. These genes form clusters comprising all five types of histones (H3, H4, H2a, H2b, and H1), and the clusters in the genome are arranged in tandem repeats in many animals (almost all invertebrates, fish, and amphibians). As for the nematodes and birds, the clusters do not form any tandem repeats but are rather dispersed throughout the genome. The mammalian (mouse and human) genome mainly contains incomplete clusters comprising the genes coding for 2–3 histone genes, which do not form any tandem repeats and are scattered over the genome [44]. Considerably less is known about the organization of histone genes in plants. The histone genes of maize, wheat, soybean, barley, and *A. thaliana* are mainly not clustered, do not form tandem repeats, and are dispersed over the genome [45,46,47]. The best studied in plants are genes coding for histone H3. The copy number of the histone H3 gene varies in different evolutionary-distant species from 13 to 16 [48]. The *A. thaliana* genome carries 15 copies of the histone H3 gene in four chromosomes [46]. It is probable that the copy numbers of the other histone genes are similar because their equimolar amounts are necessary for the nucleosome structure except for histone H1, which is needed in a half amount. Indeed, the garden pea genome has seven copies of the histone H1 gene, residing in three loci of two chromosomes [49]. Most histone genes are replication-dependent, i.e., they are expressed during the S phase of the cell cycle, but a small part is constitutively expressed during the entire interphase [50,51].

Histone genes are undoubtedly the most important housekeeping genes because they provide chromatin compaction, necessary for all eukaryotes, are involved in the epigenetic regulation of gene expression, and reside in the regions of actively transcribed chromatin. That is why the integration of target recombinant genes into the genomic regions flanking histone genes aiming to increase the expression level of transgenes is most promising. This can be the most efficient in the case of plant suspension cultures, which are represented by constantly dividing cells with almost the entire interphase consisting of the S phase.

**Figure 2:**
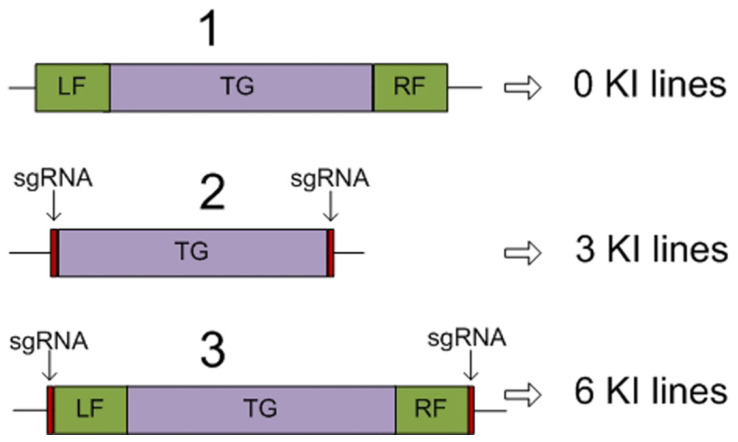
Schemes of genetic constructs for the delivery of the dIFN gene to the region of the histone H3.3 gene: LF and RF, left and right flanking sequences homologous to the corresponding regions in the *A. thaliana* genome (intergenic region upstream of the histone H3.3 gene); TG, target gene; and sgRNA, Cas9 endonuclease restriction sites, identical to the site in the intergenic region upstream of the *A. thaliana* histone H3.3 gene, to excise the construct from the plasmid in the cell; the number of obtained knock-ins (KI) are shown to the right.

We have recently inserted the target dIFN transgene (modified human γ-interferon) into the *A. thaliana* region of the intergenic spacer between the gene of microsomal signal peptidase (12 kDa subunit) and the histone H3.3 gene (At4G40040, gene *HTR5*) [20], which is constitutively expressed during the prophase of the cell cycle and is the most active in cell culture [52]. Three genetic donor templates carrying target and selective genes were constructed. The first template carried the flanking sequences homologous to the integration region; the second had no homologous flanks but was flanked with the sequences analogous to the Cas9 restriction site; and the third contained both the homologous sequences and the restriction sites (Figure 2).

Cas9 and sgRNA were contained in a separate plasmid and were delivered to cells by biolistics together with the donor plasmid in equimolar amounts. In this experiment, we recorded nine successful knock-in events in the region of histone H3.3 gene. A total of 10 biolistic shots were performed with each of 3 constructs. The first donor construct failed to give any results, whereas the second and third constructs gave three and six knock-ins, respectively (Figure 2). Thus, the third variant has proven the most effective. Concurrently, we produced five cell lines with a random insertion of the target dIFN transgene into *A. thaliana* genome. Further analysis demonstrated a considerable variation in the content of dIFN protein in the produced monoclonal cell sublines. The dIFN amount in the sublines with a randomly inserted transgene did not exceed 0.5% TSP (total soluble protein) versus the sublines with the transgene inserted into the region of H3.3 gene, where the dIFN amount varied from 1 to almost 2.5% TSP. This may be associated with somaclonal variation, frequently taking place during plant cell cultivation. In analogous experiments, a high variation in the expression level was observed during targeted gene insertion in rice [53]. However, the very amount of 2.5% TSP demonstrates a high efficiency of the strategy of inserting the transgene into the genomic regions with high transcriptional activity. RT-PCR assay of the produced cell sublines failed to show any correlation between the content of dIFN protein and the amount of the corresponding mRNA, which requires additional study [16].

### 3.4. Genes of Actin, Tubulin, and Ubiquitin

Actin and tubulin, forming the microfilaments and microtubules, are the major components of the cell cytoskeleton. *A. thaliana*, as well as rice and sorghum, has 8–10 considerably diverged copies of the actin gene scattered over four chromosomes. *Medicago truncatula* contains only 4 actin genes and maize, 21 genes [54,55]. The situation with the tubulin genes is analogous: *A. thaliana* has 12 tubulin genes (4 coding for α-tubulin and 8, for β-tubulin) residing in four chromosomes; only 2 β-tubulin genes are tightly linked [56]. Not all variants of actin and tubulin are equally expressed in different organs and tissues: some genes are more active in somatic organs and others in generative organs [55,57]. See reviews [55,58] for comprehensive data on the structure and regulation of plant actin and tubulin genes. The 5′UTRs and, possibly, introns of these genes may be promising for integration of target recombinant genes.

Ubiquitin is a short protein (76 amino acid residues), able to covalently bind with its C-terminal residue (Gly) to the side ε-amino group of Lys residues of any proteins, thereby forming a Y-shaped structure. In its conservation, ubiquitin is comparable to histone H4: ubiquitins of fungi, animals, and plants differ in 2–3 amino acid substitutions [59]. The effect of protein ubiquitination is not reduced to only well-known proteasome degradation of proteins; ubiquitin is involved in the regulation of manifold processes in the cell, including the regulation of gene transcription by binding to histones and transcription factors [60,61].

Ubiquitin genes are unique in their organization: these genes are always hybrid, and the part coding for ubiquitin is attached in a head-to-tail manner (without an intergenic spacer) to the part coding for either some other protein or another ubiquitin copy (polyubiquitin genes). In total, the *A. thaliana* genome has 12 ubiquitin genes; 5 of them are polyubiquitins (containing 3–6 repeats coding for elementary ubiquitin); 5 are fused with the regions coding for 3 different proteins (L40, S27a3, and S27a1) of the small and large ribosomal subunits; and 2 genes contain the coding sequence of RUB, a ubiquitin-like protein [59,60]. The hybrid transcript of these genes is translated as whole and post-translationally processed. Then it is cut by ubiquitin-specific proteases (deubiquitinases), which recognize Gly75–Gly76 ubiquitin residues; note that any residues except for Pro can follow in the sequence. This property of deubiquitinases was used for the construction of the proteins with new specified N-terminal structures [59,62,63] as well as for a successful increase in the expression level of target genes fused with three prime sequence of the ubiquitin gene [64]. See reviews [59,60,61] for the detailed structure of the genes coding for ubiquitin and its role in the regulation of cell processes. A ubiquitin promoter has been widely used to drive the transferred genes expression, and in many studies, it exhibited higher efficiency than a CaMV 35S promoter [65,66]. Our approach actually involved the use of not only one promoter of the ubiquitin gene but the entire genomic environment of this gene, using this region of the genome for insertion. Taking into account the unique specific features of ubiquitin genes and the capabilities of deubiquitinases, it appears feasible to transfer target recombinant genes not only to the flanking regions of ubiquitin genes, but also to the distal 5′-regions of a gene fused with ubiquitin, and in this case the target gene does not need its own promoter, because transcription will start from a ubiquitin promoter comparable in efficiency to the CaMV 35S promoter.

Since the families of actin, tubulin, and ubiquitin genes are represented in plants by rather high numbers of considerably diverged copies transcribed with different intensities in different tissues, it looks reasonable to preliminarily assay these genes by RT-PCR in order to detect the most transcriptionally active regions containing them.

Another approach to the detection of actively transcribed plant genomic regions consists in the production of a large number of random insertions of a reporter gene into the genome followed by selection of the cell lines displaying the most intensive expression of this gene. The subsequent PCR and sequencing of the regions encompassing the reporter gene in the most productive cell lines will allow the most transcriptionally active genomic regions to be detected. Then, the CRISPR/Cas9 toolkit can be used to integrate the genes of interest into these particular active regions in a targeted manner [6].

## 4. Other Approaches Providing Targeted Delivery of Transgenes to Previously Characterized Regions of the Plant Genome

In standard protocols for the transformation of plant cells, the transgene is delivered to them either using an agrobacterial infection or bioballistically. This results in the random integration of one or more copies of the transgene into the genome, some of which may adversely affect the plant phenotype and productivity. Therefore, it is necessary to obtain a large number of transformants and then select the most promising in terms of viability and transgene yield [67,68]. In contrast, inserting a transgene into a previously characterized region of the genome reduces the possibility of negative consequences of the integration and increases the likelihood of obtaining plants with the desired properties [69]. In recent years, many methods for targeted delivery of transgenes into the plant genome have been developed [12]. Here we will focus only on methods based on the use of CRISPR/Cas tools.

In 2014, the in planta gene targeting (IPGT) method was used for targeted insertion of the reporter gene into the *A. thaliana* genome [70]. The essence of the method is that Cas9 simultaneously produces DSB in the target site and in the template plasmid, freeing the linear cassette with the transgene, which is then integrated into the DSB in the host genome. A similar strategy was applied to insert the GFP gene into the *A. thaliana* genome using template plasmid and Cas9-sgRNA plasmid. It is noteworthy that although the integration frequency was less than 1%, the insertion did not contain selective markers [71].

Another approach to increase the efficiency of IPGT induction—sequential transformation—was tested in *A. thaliana* [72]. First, an *A. thaliana* line stably expressing Cas9 was obtained, and then it was transformed with a plasmid carrying sgRNA and the GFP reporter gene. In this work, the frequency of targeted integration reached 6–9% [72]. Another method for increasing the frequency of target integration is based on the use of Cas9 in combination with viral replicons from the geminivirus, which can significantly increase the amount of template DNA in the plant cell. This method has been successfully applied to tomatoes [73], potatoes [74], wheat [75], and rice [76]. Targeted gene insertions, but without the use of a viral replicon, have been successfully obtained using biolistic bombardment on several other plants, including maize [77] and soybeans [78].

An interesting approach was used in the work on rice *Oryza sativa* [15,79]. It consisted in a preliminary search for genome regions that withstand a large number of mutations without a visible effect on the plant phenotype and yield. Further, targeted integration of the desired transgenes into these regions of the genome was performed. Using the CRISPR/Cas9 toolkit, the authors inserted two genes for carotenoids biosynthesis into two such “safe harbors” sites and obtained rice with a high content of carotenoids in seeds, so called “golden rice” [15]. It is noteworthy that, in this work, the expression cassettes, in addition to the sgRNA recognition sites ensuring their excision from the plasmid, contained flanking sequences homologous to the insertion site. In our work on transgene integration into the region of the histone H3.3 gene, a template with homologous flanks also showed the highest frequency of insertion [16], which apparently indicates that insertion occurs, not only through the NHEJ mechanism, but also through the HDR homologous repair mechanism. However, there is another explanation for this fact. Recently, it was shown in rice that the normally very low HDR frequency can be greatly increased by the formation of two tandemly repeated elements, the so-called TR-HDR strategy. In this case, the incorporation of the template occurs mainly according to the NHEJ mechanism, and the flanks of the template and the insertion region form a tandem repeat, which stimulates the replacement of sequences of insertion region with homology flanks from the template [79].

## 5. Activation (Euchromatization) of Certain Genomic Regions

**Figure 3:**
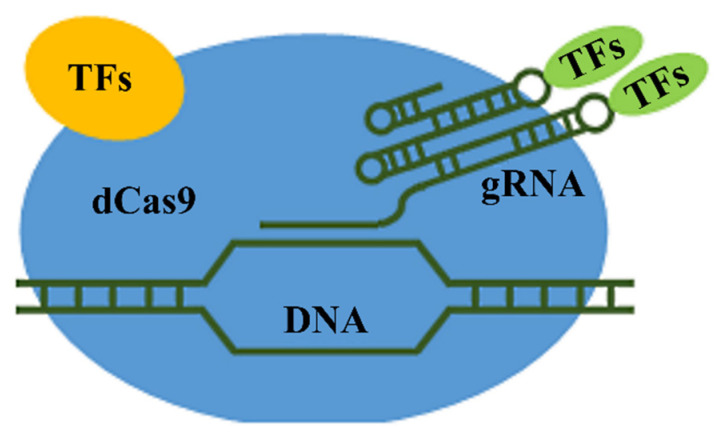
Basic scheme of targeted transcription activation in specified genomic regions: TFs (yellow oval), transcription activation factors attached to dCas9; and TFs (green oval), transcription activation factors attached to gRNA.

A completely new approach to an increase in the expression level of recombinant proteins in plants based on CRISPR/Cas9 technologies has been recently developed. This approach consists of targeted transcription activation in specified genomic regions. It relies on the use of deactivated endonucleases (dCas), which, thanks to their guide sgRNA, bind to the sequence of interest in the genome but are unable to make a double-strand break. Such a dCas fused to the factors activating transcription delivers them to the required genomic region. This approach was for the first time used with human cells [80,81], and then the CRISPRa system was applied to plant cells using dCas9 with five tandemly repeated VP64 transcription factors (dCas9–VP64) [82,83]. Three new systems simultaneously carrying several transcription factors—dCas9–SunTag [84], dCas9–TV [85], and dCas9–EV2.1 [86]—were designed to increase the transcription activation effect. The dCas9–EV2.1 system displayed the highest efficiency: it induced a 3–13-fold increase in the transcription intensity of different genes with different promoters [86]. In the dCas9–SunTag system, dCas is fused with a tandem array of GCN4 peptides, which attract VP64 transcriptional activators [84]. The dCas9–TV system utilizes the attachment of six transcription activator-like effector (TALE) copies united with a VP128 activator to dCas9, 6×TALE–VP128 (TV) [85]. Another approach is used in the dCas9–EV2.1 system: the guide sgRNA is attached to the anchor sites for the VPR transcriptional activator, VP64–p65–Rta [86]. Later, two additional systems were designed: one is CRISPR–Act2.0, based on the fusion of dCas9 with the VP64 and EDLL transcription factors and the attachment to sgRNA of two MS2-binding aptamers, recruiting additional VP64 factors, and the other, mTALE-Act, carrying both TALE and VP64 activators [87]. Figure 3 shows the basic scheme of transcription activation.

All these transcription activation systems give a very wide range of the degree of activation depending on the genes to be activated. In particular, dCas9–VP64 elevates 7-fold the transcription level of the *A. thaliana* PEP1 gene versus a 200-fold increase in the transcription of the FIS2 gene. The mTALE–Act system increases the transcription level of both genes approximately 30-fold, while CRISPR–Act2.0 gives 30–45- and 1500-fold increases, respectively, for PEP1 and FIS2. Interestingly, CRISPR–Act2.0 is able to activate rather long chromatin regions comprising up to four genes [87].

The system CRISPR–Act3.0, based on CRISPR–Act2.0, has been designed just recently: this system is supplemented with the elements of SunTag (10 tandem repeats of GCN4 peptide) and TALE (2 TAD activator repeats). The new third generation system has been tested with rice *OsGW7* and *OsER1* genes and demonstrated a 10-fold higher efficiency as compared with the second-generation system, CRISPR–Act2.0. The transcription level of *OsGW7* grew 250-fold and of *OsER1*, 100-fold; note that the region of this effect covered up to seven adjacent genes. An analogous system has been constructed involving dCas12b and also displays impressive results [88].

Although all these systems for increasing the transcription level have been so far tested using the own genes of plants, they can be undoubtedly used for increasing the expression level of foreign target genes and even a set of several genes, which can create new metabolic pathways in the plant cell.

## 6. Spatial Organization of Chromatin and Transcriptional Activity

The eukaryotic cell faces the difficult task of accommodating a considerable amount of DNA within its small nucleus. The genome of several plant species is 50-fold larger as compared with the human genome. In addition to packing into nucleosomal structures, the eukaryotic genome forms the structures of higher orders [89,90]. The chromatin is arranged in the nucleus in a nonrandom manner; each chromosome occupies its own chromosome territory, which influences the availability of the genes for different factors and their expression [91,92]. In turn, chromatin can also form topologically associated domains that divide large regions of the genome into distinctly defined autonomously regulated regions [93,94].

In addition, the genome is arranged as local chromatin loops, which can considerably influence the transcription level of genes [93,95]. These loops can be large and unite the genes rather distant from one another, allowing them to exchange different factors involved in expression regulation [96]. These loops can be short, covering a single locus, and provide the chromatin interaction within a single gene, which makes it possible to most dynamically regulate its expression [97].

**Figure 4:**
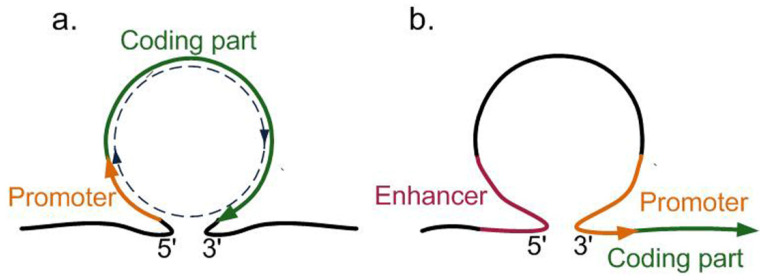
Chromatin loops increasing the transcriptional activity of genes: (**a**) the loop between 5′UTR and 3′UTR, allowing RPol II to move in a circle; and (**b**) the loop between a distant enhancer and a promoter.

Short chromatin loops can be of manifold types, can cover different gene regions, and provide different types of transcription regulation, increasing or decreasing the transcription intensity of genes, initiating or prohibiting the reverse reading of noncoding RNA or antisense RNA, and leading to an alternative splicing [98]. In light of the topic of this review, the gene loops that lead to an increase in the transcription of a gene they contain are most interesting to us. First and foremost, this is the loop where the 5′UTR and 3′UTR of the same gene are brought close to each other (Figure 4a). The loop of this type leads to the formation of a separate isolated transcriptional unit from the promoter to the transcription termination site (TTS). This allows RNA polymerase II to work more efficiently: once having reached TTS, it can immediately bind the promoter and further move in the circle [97,98,99].

At least five cases are known in plants when the chromatin loops between 5′UTR and 3′UTR are formed [97]. The sunflower *Helianthus annuum* gene *HaWRKY6* forms a gene loop in the cotyledon cells, which ensures a high tissue-specific expression thanks to the recirculation of RNA polymerase II. This gene almost does not express in the cells of leaves [100]. The loop in the sunflower gene *FLC* (flowering locus) disappears after 2 weeks of incubation in the cold, which causes a decrease in the *FLC* transcription and slows down the switch to flowering [101]. In *A. thaliana*, the gene loops enhancing transcription have been found in three genes: *IPT3*, *IPT7*, and *TFL1* [102,103]. Another type of the loops that elevate the transcription level is the loops between a distal enhancer and a promoter (Figure 4b); the loops of this type have been observed in *Zea mays* [103] and *A. thaliana* [104].

The mechanisms underlying the formation of chromatin loops are vague and require further in-depth studies. In several cases, the interaction between protein transcription factors contributes to the formation of loops [104,105]. The loop formation also depends on the balance between the histone H3 methylation and acetylation in the region and DNA methylation [101,106]. Short interfering RNAs are also involved in this process [97,100,101,107]. Unfortunately, now, existing molecular methods do not allow us to artificially create such loops increasing the level of gene transcription. It is important to realize that transcriptional activity is to a considerable degree determined by the spatial organization of a chromatin region.

## 7. Conclusions

Considering various examples of site-specific delivery of target genes using the CRISPR/Cas system, it becomes obvious that this work should be preceded by a search for optimal areas in the genome for targeting genetic engineering tools to them. As such, researchers consider genomic “safe harbors”, the choice of which directly depends on the task set by the researcher. When improving any characteristics of varieties of important agricultural crops, one of the main criteria is the preservation of the characteristics of the variety created by the previous efforts of breeders.

The choice of search criteria for GSH for the field of biotechnology of cultivated plant cells, in our opinion, is determined primarily by the choice of such regions in which integration of the target gene will provide the highest possible yield of recombinant protein. On the one hand, housekeeping gene loci can be such areas. The peculiarity of the organization of such regions is associated with the involvement of many transcription activation factors. Although, as it was shown by the first attempts to deliver a target gene to areas of housekeeping genes, not all of them may be available for genomic editing. On the other hand, such regions can be identified by evaluating the loci of the random transgene integration into the plant genome and determining the regions in which the expression of the target gene and the yield of the recombinant protein are maximum.

Works on chromatin modification and the creation of “artificial” GSHs that are attractive for target transgene delivery also seem promising. The spatial organization of chromatin is an efficient regulator of many aspects of transcription. The chromatin in the nucleus is nonrandomly arranged, forming various loops that regulate and modulate its activity. The 3′UTRs and 5′UTRs of the actively transcribed genes are brought into proximity, which considerably optimizes the work of RNA polymerase II, allowing it to move in a circle. The mechanisms and methods underlying the formation and maintenance of such loops are rather vague; however, this side in the chromatin organization and function should not be overlooked.

## Figures and Tables

**Table 1:** Housekeeping genes of *A. thaliana* most promising for insertion of the genes coding for target recombinant proteins.

Housekeeping Genes	Copy Number in Haploid Genome	Arrangement in Genome	Distribution over Chromosomes (Loci)	Transcribed by *	Phase of Activity
35S rRNA genes	750	Tandem clusters 18S, 5.8S, and 25S rRNAs	Two loci in two chromosomes	RPol I	Constitutively active in the interphase
5S rRNA genes	1000	Tandem repeats	Four loci in three chromosomes	RPol III	Constitutively active in the interphase
tRNA genes	10–20 (of each tRNA)	In part, form tandem repeats; genes coding for different tRNAs are dispersed over the genome	All chromosomes	RPol III	Constitutively active in the interphase
Histone genes	15 (of each of the five histones)	Mainly not clustered and do not form tandem repeats	In four chromosomes (histone H3)	RPol II	S phase; part of copies is constitutively active in the interphase
Actin genes	8–10	Dispersed over the genome	In four chromosomes	RPol II	Constitutively active in the interphase
Tubulin genes	12	Dispersed over the genome	In four chromosomes	RPol II	Constitutively active in the interphase
Ubiquitin genes	12; 5 of them, code for polyubiquitin (3–6 repeats)	Dispersed over the genome	All chromosomes	RPol II	Constitutively active in the interphase

* RPol, RNA polymerase.
